# A Randomized, Double-Blind, Placebo-Controlled Phase I Study to Evaluate the Safety, Tolerability, and Immunogenicity of an Outer Membrane Vesicle (OMV) Platform-Based Vaccine Administered Intranasally to Healthy Adults

**DOI:** 10.3390/vaccines14070575

**Published:** 2026-06-29

**Authors:** Heleen Kraan, Anne van der Geest, Dinja Oosterhoff, Corine Kruiswijk, Peter Soema

**Affiliations:** 1 AdJane BV, Thijsseweg 11, 2629 JA Delft, The Netherlands; 2 Intravacc BV, Antonie van Leeuwenhoeklaan 9, 3720 AL Bilthoven, The Netherlands; 3 Athena Institute, Vrije Universiteit, Boelelaan 1105, 1081 HV Amsterdam, The Netherlands

**Keywords:** OMV, adjuvant, intranasal vaccine, SARS-CoV-2, *Neisseria meningitidis*, vaccine platform, mucosal immunity

## Abstract

**Background:** The COVID-19 pandemic exposed critical gaps in pandemic preparedness and highlighted the need for vaccine platforms capable of rapid adaptation. Outer membrane vesicle (OMV)-based platforms utilizing vesicles derived from genetically detoxified *Neisseria meningitidis* serogroup B (Nm-nOMV) represent a promising plug-and-play approach. **Methods:** This Phase I, first-in-human, randomized, double-blind, placebo- and OMV-controlled trial, evaluated safety, tolerability, and immunogenicity of intranasally administered OMVs combined with SARS-CoV-2 Spike protein in healthy SARS-CoV-2 seropositive adults aged 18–55 years. Forty participants were enrolled across two cohorts: a low-dose cohort receiving 140 μg OMV/70 μg Spike (OMV + Spike, *n* = 13; OMV alone, *n*= 3; Placebo, *n* = 5) and a high-dose cohort receiving 280 μg of OMV/140 μg of Spike (OMV + Spike, *n* = 13; OMV alone, *n* = 3; Placebo, *n* = 3), administered on Days 1 and 22. Safety was assessed through adverse events, vital signs, laboratory parameters, ECG, and pulse oximetry. Immunogenicity was evaluated via systemic SARS-CoV-2 neutralizing antibodies, antigen-specific antibodies (IgG and IgA), and mucosal antibodies (IgA in nasal wash). **Results:** Intranasal administration of OMVs combined with SARS-CoV-2 Spike protein was safe, well-tolerated, and immunogenic. No serious adverse events were reported, and adverse events were predominantly mild and transient. Dose-dependent increases in systemic and mucosal immune responses were observed, with statistically significant enhanced serum IgG and nasal wash IgA antibodies in the high-dose group. **Conclusions:** The current clinical data confirm key aspects of the preclinical profile, which demonstrate the potential of the Nm-nOMV platform as a strong adjuvant for mucosal vaccines. These findings support the broader application of the Nm-nOMV vaccine platform in pandemic preparedness.

## 1. Introduction

The COVID-19 pandemic exposed critical deficiencies in global pandemic preparedness and underscored the urgent need for versatile vaccine platforms capable of rapid deployment against rapidly spreading emerging pathogens. While vaccines were developed at exceptional speed using platforms such as nucleoside-modified mRNA (Comirnaty^®^, Pfizer, New York, NY, USA; Moderna Cambridge, MA, USA), non-replicating viral vectors (Vaxzevria^®^, AstraZeneca, Cambridge, UK; Jcovden^®^, Vaccines & Prevention, Leiden, The Netherlands), and recombinant nanoparticle technology (Nuvaxoid^®^, Novavax, Gaithersburg, MD, USA), each platform demonstrated distinct advantages and limitations, including stability, manufacturability, and immunogenicity [1]. Despite substantial timeline compression during the COVID-19 pandemic, conventional development of vaccines may still be challenged by fast-evolving viruses, as their rapid mutation rates might outpace vaccine efficacy over time [2].

Plug-and-play vaccine platforms represent a transformative approach by leveraging established backbones, thereby exploiting pre-validated manufacturing processes and established safety dossiers. This approach reduces the need for repetitive toxicology assessments and good manufacturing practice validation, thereby streamlining regulatory authorization and enabling accelerated scale-up against diverse emerging pathogens [3]. In this context, outer membrane vesicle (OMV)-based vaccine platforms represent a promising approach with demonstrated versatility and broad applicability [4]. OMVs are naturally secreted, non-infectious vesicles derived from Gram-negative bacteria that present surface antigens in conformations readily recognized by the immune system and simultaneously activate innate immune responses through multiple pathogen-associated molecular patterns (PAMPs), including lipopolysaccharide (LPS) and lipoproteins [5,6]. Moreover, OMV-based vaccines have demonstrated the capacity to induce both systemic and mucosal immune responses, making them relevant in the application towards pandemic preparedness [6,7]. 

In line with this, increasing efforts are directed towards intranasal vaccines against respiratory pathogens, including SARS-CoV-2, using platforms such as live attenuated viruses, viral vectors, and recombinant protein-based formulations to elicit mucosal immunity at the site of infection [8,9,10]. Among them, viral vector-based vaccine platforms represent one of the most widely explored technologies for intranasal vaccine development. However, only a limited number of intranasal vaccines have been approved for human use, and clinical data indicate that several viral vector-based candidates induce relatively weak mucosal immune responses and may be affected by pre-existing vector immunity and biosafety constraints [11]. Within this landscape, OMVs are particularly relevant due to their intrinsic adjuvanticity and capacity to induce both systemic and mucosal immune responses. Nevertheless, few OMV-based candidates have advanced to human clinical trials, underscoring the need for further studies on their safety and efficacy in humans [11].

An OMV vaccine platform based on genetically modified *Neisseria meningitidis* serogroup B (Nm-nOMV platform) has been developed and refined over more than three decades to optimize safety and immunogenicity characteristics, with stability demonstrated for at least 2.5 years in ongoing studies under standard refrigerator conditions. In contrast to classical detergent-extracted OMVs—in which deoxycholate removes the majority of LPS and associated lipid components—the Nm-nOMV platform employs a detergent-free production process, preserving the native outer-membrane composition, including lipoproteins that contribute to immunostimulation [12,13]. Three complementary genetic modifications of the strain further enhance the platform’s safety, yield, and deployment flexibility: deletion of *lpxL1* yields a penta-acylated lipid A with substantially reduced reactogenicity [14]; deletion of rmpM increases spontaneous vesiculation and therefore manufacturing yield [12]; and removal of the immunodominant PorA outer membrane protein limits immunity against the OMV, preserving the platform for repeated and heterologous use [13].

The Nm-nOMV platform supports multiple antigen presentation strategies. As a standalone adjuvant it can be co-administered with exogenous antigens to enhance antigen-specific and protective immune responses. Alternatively, antigens can be chemically conjugated to OMV surfaces enabling multivalent presentation, or OMVs can be used as a vehicle for heterologous antigen expression directly within the outer membrane, creating a single-component construct that combines targeted antigen presentation with the intrinsic adjuvant properties of the OMV. This flexibility allows adaptation to different antigen types and manufacturing requirements.

Preclinical mouse studies demonstrated robust cellular and humoral immune responses against SARS-CoV-2 Spike protein when co-administered with Nm-nOMVs, supporting the rationale for clinical evaluation [6]. This first-in-human phase 1 study evaluated the safety, tolerability, and immunogenicity of intranasally administered Nm-nOMVs combined with stabilized prefusion Spike protein. The primary objective was to assess safety and tolerability through comprehensive monitoring of adverse events, vital signs, laboratory parameters, pulse oximetry, and cardiac function. Secondary objectives included the evaluation of immunogenicity via SARS-CoV-2 neutralizing antibodies, serum IgG and IgA responses, and mucosal IgA in nasal wash samples. This study provides the first clinical evaluation of the Nm-nOMV platform, establishes clinical proof-of-concept, and lays the foundation for its broader application in pandemic preparedness. 

## 2. Materials and Methods

### 2.1. Study Design and Participants

This phase I, first-in-human, randomized, double-blind, placebo-controlled, and OMV-controlled clinical trial was conducted in a dedicated clinical research unit (CRU) in Australia to evaluate the safety, tolerability, and immunogenicity of intranasally administered Nm-nOMVs (hereafter referred to as OMV) combined with SARS-CoV-2 Spike protein in healthy adult male and female participants. The study was conducted in accordance with the Declaration of Helsinki and according to Good Clinical Practice (GCP). No patient or public involvement took place during the trial design, conduct or reporting. The protocol, protocol amendments, Informed Consent Form (ICF), Investigator Brochure, and other relevant documents (e.g., advertising material) were reviewed and approved by the Human Research Ethics Committee (HREC) before the study was initiated. The study was registered with ClinicalTrials.gov on 1 November 2022 under the identifier: NCT05604690.

**Figure 1:**
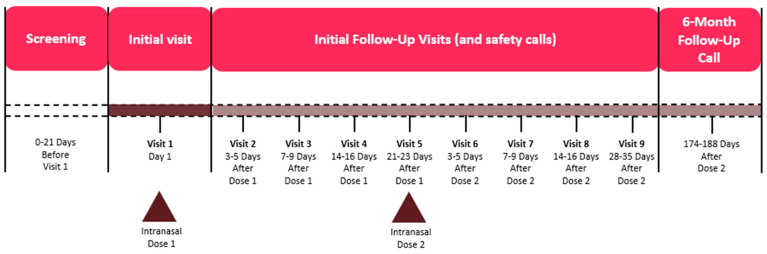
Study design and visit schedule. Timeline showing screening, vaccination visits, and follow-up (FU) assessments. Participants receive intranasal doses on day 1 and day 22 (±1 day), with safety monitoring and immunogenicity assessments conducted at predefined intervals up to 6 months post-vaccination.

Forty participants were enrolled across two cohorts. Cohort 1 received a low dose of OMVs mixed with SARS-CoV-2 Spike antigen (hereafter referred to as Spike) (140 μg of OMVs and 70 μg of Spike protein), OMV alone (140 μg), or placebo. Cohort 2 received a corresponding high-dose formulation (280 μg of OMVs and 140 μg of Spike protein), OMV alone (280 μg), or placebo. Each participant received doses on Day 1 and Day 22; each dose consisted of 0.4 mL total (4 times 0.1 mL, alternating per nostril per dosing procedure), administered in supine position. Both cohorts followed a sentinel dosing strategy, with four sentinel participants dosed prior to the rest of the cohort and safety and tolerability data reviewed at least four days later by the Safety Review Committee before dosing of the remaining participants. The four sentinel participants represented each of the randomized treatment groups in a 2:1:1 ratio (OMV + Spike, OMV, and placebo, respectively). Participants were discharged approximately 4 h after dosing if no clinically significant safety findings were observed, and follow-up assessments were performed on Day 4 (±1 day) and Day 15 (±1 day), 3 to 5 days and 14 to 16 days after dose 2, at 28 days (window 28 to 35 days), and at 181 days (±7 days) post-dose 2. Safety calls were conducted 7 to 9 days after each dosing visit. An overview of the study design is provided in Figure 1.

The study enrolled participants who were seropositive for SARS-CoV-2, defined as individuals who had received a SARS-CoV-2 vaccination or had confirmed prior exposure at least four months before enrolment, or who tested seropositive for IgG antibodies using any serological assay authorized under Emergency Use Authorization by the U.S. Food and Drug Administration (FDA). Eligible participants were adults aged 18–55 years and in good general health with no clinically significant findings based on medical history, physical and neurological examination, vital signs (including systolic and diastolic blood pressure (BP), temperature, and pulse rate (PR)), 12-lead electrocardiogram (ECG), and clinical laboratory evaluations. Participants were required to have a body mass index (BMI) between 18.0 and 32.0 kg/m^2^ and a body weight of ≥50.0 kg for males and ≥45.0 kg for females. A full overview of all inclusion and exclusion criteria is provided in Supplementary S1. All participants provided written informed consent before performing any study-specific procedures, confirming their understanding of the study requirements and their willingness to comply with all protocol restrictions.

### 2.2. Randomization and Blinding

Eligible participants were randomized according to a simple, computer-generated randomization schedule prepared by an independent biostatistician using validated statistical software, SAS^®^ version 9.2 (SAS Institute, Cary, NC, USA). Following informed consent, each participant was assigned a unique screening number and, upon confirmation of eligibility, a sequential randomization number prior to first dosing. Participants within each cohort were randomized to receive OMV + Spike, OMV, or placebo according to the predefined allocation schedule.

This study was conducted in a double-blind manner, with the sponsor, investigators, medical monitor, study personnel, and participants remaining blinded to treatment allocation. Unblinded pharmacy personnel (or other qualified site staff) were responsible for preparation of the investigational product, while designated unblinded clinical staff oversaw treatment administration; these individuals were not involved in study assessments to maintain blinding integrity.

### 2.3. Vaccines

The vaccine candidate comprises two components, OMVs and a SARS-CoV-2 Spike protein. The OMV (manufactured at Intravacc BV, Bilthoven, The Netherlands) was formulated in 0.01 M Tris, 0.09 M sucrose. The SARS-CoV-2 Spike protein (manufactured at ExcellGene SA, Monthey, Switzerland) was formulated in phosphate-buffered saline. OMVs were produced by a modified *Neisseria meningitidis* serotype B (NmB) strain. The starting material for the vaccine strain construction is NmB strain H44/76 HI5, which is a spontaneous PorA deletion mutant [15]. Several other genetic modifications were made to increase safety during handling in the manufacturing process, increase blebbing of the outer membrane to increase OMV yields, and reduce lipopolysaccharide (LPS) endotoxicity. The Spike antigen is derived from the SARS-CoV-2 Wuhan strain in a prefusion state with 6 proline substitutions and produced in Chinese Hamster Ovary cells. They are supplied as sterile suspensions in separate vials, which are mixed just prior to administration to generate the vaccine candidate.

### 2.4. Procedures

Participants were administered their assigned treatment (OMV + Spike, OMV, or Placebo) intranasally via an intranasal Mucosal Atomization Device (Teleflex Medical BV, Vianen, The Netherlands) fitted via Luer lock to a syringe. At each dosing date, the participant rested in a supine position for at least 5 min prior to administration. A volume of 0.1 mL of the assigned treatment was administered to the participant’s first nostril in a standardized procedure involving brief breath-holding after inhalation, with exhalation via the mouth. The procedure was repeated four times alternating each nostril, resulting in a total administered volume of 0.4 mL per dose. The entire administration sequence was completed within approximately 1 min, although minor variations were not considered protocol deviations. Participants returned for a second dose of their assigned treatment on Day 22 (±1 day), where the same administration procedure was repeated. All doses were administered at the clinical research unit by a designated member of the clinical team.

Blood samples for immunogenicity analysis and cellular response, along with nasal washes for SARS-CoV-2 IgA assessment, were collected according to the site’s standard operating procedures. Study procedures were completed as delineated in the Schedule of Assessments in Supplementary S2. Three minor protocol amendments were made, as described in Supplementary S3.

Safety assessments, including pregnancy testing, physical and neurological examinations, vital signs, viral testing, hematology, biochemistry, urinalysis, and 12-lead ECG, were performed according to the site’s standard operating procedures and are provided in Supplementary S4. Additionally, participants were provided with a paper diary for a period of approximately 2 weeks after each dose of assigned treatment (from Day 1 to Day 15 [±1 day] and Day 22 [±1 day] to Day 36 [±2 days]). The diary consisted of a set of defined questions querying the participant’s experiences and any reactions to the study treatment, as well as any concomitant medications taken. If the participant had experienced any adverse reactions (such as headache, fever, fatigue, etc.), they were instructed to describe the event, the time it occurred, and when (and if) the event resolved. The Investigator or designee then reviewed these diary entries and the Investigator determined if there were any events which met the definition of an Adverse Event (AE), and performed follow-up as necessary, either to provide medical advice or to attain further information on an entry.

### 2.5. Outcomes

The primary outcome of this study was to evaluate the safety and tolerability of intranasal administration of OMV + Spike in healthy participants, assessed by the incidence, severity, and duration of AEs including systemic and local, as well as signs of common allergic reactions, and signs of severe allergic reactions; incidence, severity, and duration of serious adverse events (SAEs); and incidence of clinically significant vital signs (systolic BP, diastolic BP, PR, and temperature), safety laboratory (blood chemistry, hematology, and urinalysis), pulse oximetry, or ECG findings. The secondary outcomes of the study included the evaluation of the immunogenic response of intranasal administration of OMV + Spike in healthy participants, assessed by SARS-CoV-2 Neutralizing Antibodies (NAbs), Immunoglobulin IgA and IgG in serum, and IgA in nasal wash at predefined time points before and after dosing.

### 2.6. Immunological Readout

Neutralizing antibody titers against SARS-CoV-2 were determined by Viroclinics (Rotterdam, the Netherlands) using a live virus neutralization assay. Briefly, serial dilutions of heat-inactivated serum were incubated with a standardized preparation of SARS-CoV-2 infectious units. The mixture was subsequently added to a confluent monolayer of Vero-E6 cells (CRL-1586, ATCC) and, following incubation and fixation, viral infection was quantified by immunostaining. Total IgG and IgA antibodies directed against specific SARS-CoV-2 antigens, including the receptor-binding domain (RBD), Spike protein, and nucleoprotein, were quantified by Viroclinics using the Meso Scale Discovery (MSD) Platform. Antibody concentrations were expressed relative to an internal reference standard.

### 2.7. Statistical Analysis

The sample size for the study was not based on statistical hypothesis testing. No data regarding variances, mean values, or event rates were available for this vaccine, as this was the first-in-human trial. Therefore, the sample size was based on the literature from other vaccines against SARS-CoV-2. Based on these trials [16,17,18] it was anticipated that the sample size of at least 36 participants was sufficient to meet the study objectives.

The Safety Population was defined as all randomized participants who received at least one dose of study drug, based on the actual treatment received. All safety analyses were performed using the Safety Population. The Immunogenicity Population consisted of all participants who received at least two doses of study drug, had at least one immunogenicity assessment at baseline, and had at least one immunogenicity assessment post-baseline. For the immunogenicity analyses, additional statistical testing was performed using the Friedman test in R-Studio (version 4.5.3). GraphPad Prism (version 11.0.0) was used for data visualization and graph generation.

## 3. Results

### 3.1. Demographic and Other Baseline Characteristics

**Figure 2:**
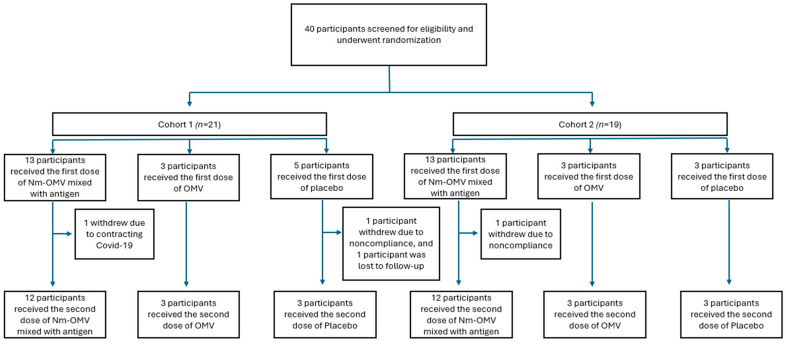
Flow diagram for recruitment, enrolment, and completion of trial.

The flow of participants in the study is shown in Figure 2. Of 66 individuals screened, 40 were enrolled and randomized across two cohorts, each comprising the OMV + Spike, OMV alone, or the placebo group. Participant recruitment started on 16 November 2022 and was completed on 15 January 2024. In cohort 1, three individuals (14.3%) withdrew before the second dose; the remaining 18 participants (85.7%) completed the study.

In cohort 2, one individual (5.3%) withdrew before the second dose, and the remaining 18 participants (94.7%) completed the study. The Immunogenicity Population comprised 12 (92.3%) participants in the OMV + Spike group, 3 (100%) in the OMV group, and 3 (60.0%) in the Placebo group for Cohort 1; and 12 (92.3%), 3 (100%), and 3 (100%), respectively for Cohort 2.

The baseline physical and demographic characteristics of enrolled individuals are presented in Table 1.

### 3.2. Safety

In this study, analyses of safety assessments were conducted as part of the primary objective. These safety assessments included examinations of safety events, including treatment-emergent adverse events (TEAEs) by severity and relationship to treatment, adverse events of special interest (AESIs), as well as changes in vital signs, safety laboratory parameters, pulse oximetry, and ECG findings. An overall summary of TEAEs is provided in Table 2.

In Cohort 1, a total of 52 TEAEs were reported in 19 (90.5%) participants, with events distributed broadly similarly across the OMV + Spike, OMV, and Placebo groups. In Cohort 2, 29 TEAEs were reported in 14 (73.7%) participants, again with a broadly similar distribution across treatment groups. Notably, the higher-dose cohort (Cohort 2) showed fewer TEAEs overall, particularly in the OMV + Spike group, where 21 events were reported in 10 (76.9%) participants compared with 31 events in 11 (84.6%) participants in Cohort 1.

Most of the TEAEs in both Cohort 1 and Cohort 2 were mild in severity. In Cohort 2, there was one severe event of Increased Blood Creatine Phosphokinase reported, which resolved without intervention and did not result in study discontinuation or any change in study treatment. No serious TEAEs were reported in any group.

Treatment-related TEAEs were defined as TEAEs possibly, probably, or definitely related to study treatment. In Cohort 1, the majority of the 52 TEAEs were deemed definitely unrelated (19 events) or unlikely related (14 events) to treatment, while 8 events were classified as possibly related, 10 as probably related, and 1 as definitely related. In Cohort 2, out of the 29 TEAEs, 14 events were described as being unrelated to treatment, 2 events were described as being likely related, 4 events were described as being possibly related to treatment, 7 events were described as being probably related to treatment, and 2 events were described as being definitely related to treatment. Although treatment-related TEAEs were more abundant in absolute terms in the OMV + Spike group, their incidence was proportionally comparable across the OMV + Spike, OMV, and Placebo groups when adjusting for the number of participants.

A detailed overview of treatment-related TEAEs and their severity across subgroups is presented for Cohort 1 in Table 3, and for Cohort 2 in Table 4. In Cohort 1, treatment-related TEAEs were predominantly mild (Grade 1) and most frequently reported within the respiratory, thoracic and mediastinal disorders and nervous system disorder SOCs, with oropharyngeal pain, nasal congestion, rhinorrhea, and headache being the most common events. No Grade 3 treatment-related TEAEs were observed, and each Grade 2 event type was reported in one participant only. In Cohort 2, where higher doses were administered, all treatment-related TEAEs occurred in a single participant, except for headache, which was the only event reported in two participants. No Grade 3 treatment-related TEAEs were observed.

In Cohort 1, a total of 22 AESIs were reported among 11 (52.4%) participants, while in Cohort 2, 10 AESIs were reported in 7 (36.8%) participants. Notably, all AESIs across both cohorts were related to local or systemic vaccine reactions, with no events reported for severe reactions, generalized convulsion, Guillain–Barré Syndrome (GBS), acute disseminated encephalomyelitis (ADEM), anaphylaxis, vasculitis, encephalitis/encephalomyelitis, facial palsy, thrombocytopenia, or vaccine-associated enhanced disease (VAED).

Few clinically significant laboratory abnormalities were observed across hematology, coagulation, serum chemistry, and urinalysis assessments. All reported clinical laboratory findings resolved to normal by at least Visit 9, except for one elevated creatine kinase event that remained above normal but deemed not clinically significant. No clinically significant changes were noted in vital signs or pulse oximetry, and only one clinically significant ECG finding was reported, which normalized at the subsequent visit.

The data demonstrate that OMV + Spike was well tolerated in this healthy participant population, with TEAEs primarily administration-related, as evidenced by events being no more frequent in the OMV + Spike groups (Cohort 1 and 2) than in OMV or placebo groups. Notably, no dose-dependent increase in safety events was observed between the OMV + Spike dose levels of Cohort 1 and 2, which is commonly observed with therapeutic products, indicating a favorable safety profile across the tested dose range (up to 280 μg of OMV and 140 μg of Spike protein). Moreover, the comparable safety profiles of OMV alone and placebo controls further confirm the inherent safety of the Nm-nOMV platform independent of Spike antigen addition.

### 3.3. Immunogenicity Evaluations

Immunogenicity assessments were conducted as part of the study’s secondary objectives. These immunogenicity assessments consisted of the analysis of SARS-CoV-2 neutralizing antibodies (NAbs), as well as anti-SARS-CoV-2 IgA and IgG in serum and anti-SARS-CoV-2 IgA in nasal wash samples.

Serum SARS-CoV-2 NAb concentrations showed low variability across visits in the low-dose OMV + Spike group (Cohort 1), OMV, and Placebo groups, with minimal changes from baseline. In contrast, the high-dose OMV + Spike group (Cohort 2) exhibited a gradual increase baseline to Visit 9, suggesting a potential dose-dependent effect (see Supplementary S5).

Antigen-specific serum IgA (directed against Spike, S1 RBD, and nucleoprotein) assessments showed an increase from baseline to Visit 9 in the high-dose OMV + Spike group (Cohort 2), whereas in the low-dose OMV + Spike group (Cohort 1), OMV, or placebo groups exhibited low variability across visits with changes from baseline (see Supplementary S6). This finding is consistent with a potential dose-dependent effect of OMV + Spike.

**Figure 3:**
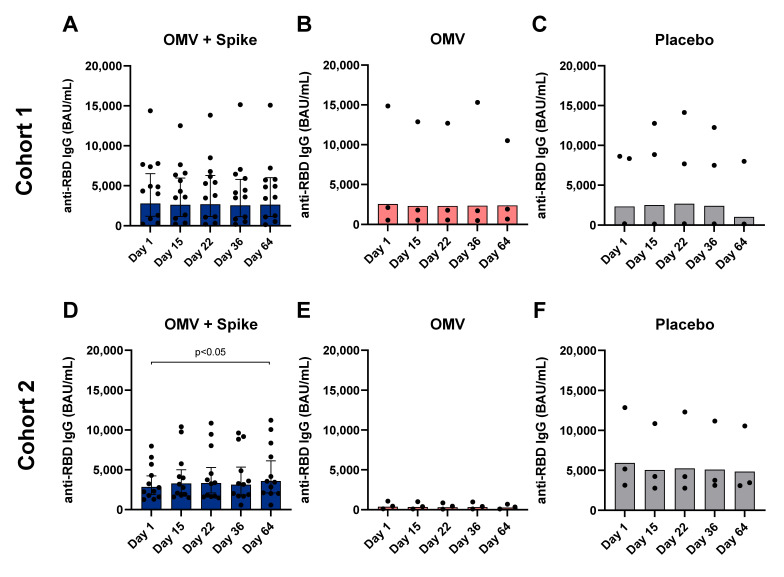
RBD-specific IgG levels measured in serum samples at different study days following vaccination with outer membrane vesicle (OMV)-based vaccine (OMV + Spike) or controls (OMV or Placebo). Serum samples were taken on day 1 (visit 1), day 15 (visit 4), day 22 (visit 5), day 36 (visit 8), and day 64 (visit 9). Bars represent geometric mean values, error bars depict 95% confidence intervals, and symbols represent measured values of individual subjects. Statistically significant differences (*p* < 0.05, Friedman test) between groups are indicated above bars. Panels (**A**–**C**) show Cohort 1 (OMV + Spike, OMV, and Placebo), and panels (**D**–**F**) show Cohort 2 (OMV + Spike, OMV, and Placebo).

As shown in Figure 3, for the assessment of RBD-specific serum IgG, similar patterns were observed in Cohorts 1 (Figure 3A–C) and 2 (Figure 3D–F), with an increase from baseline to Visit 9 in the OMV + Spike group for Cohort 2, while OMV- and placebo groups for both Cohorts exhibited low variability across visits (see Supplementary S7). Notably, the change from baseline to Visit 9 was statistically significant in Cohort 2 (Friedman test, *p* < 0.05) (Figure 3D), thereby further supporting a potential dose-dependent response to OMV + Spike, in line with the trends previously observed for antigen-specific IgA levels and NAbs.

**Figure 4:**
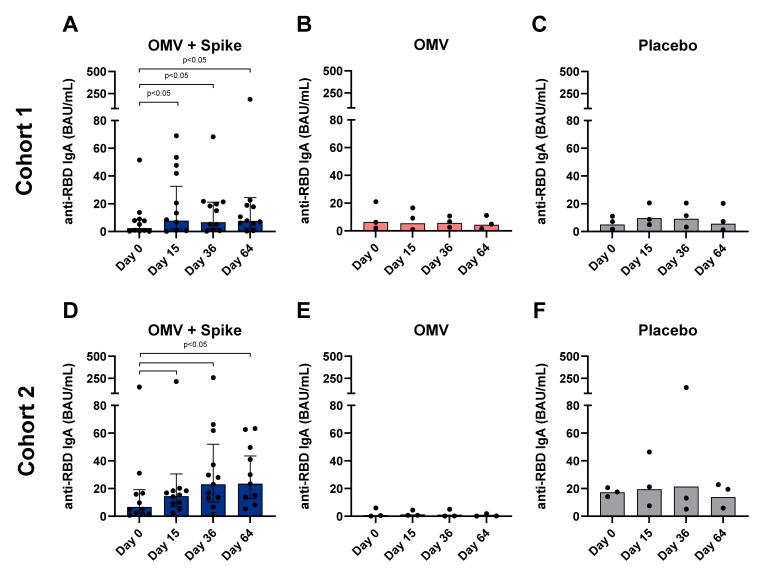
(**A**–**F**): RBD-specific IgA levels measured in nasal washes at different study days following vaccination with outer membrane vesicle (OMV)-based vaccine (OMV + Spike) or controls (OMV or Placebo). Nasal washes were conducted on day 1 (visit 1), day 15 (visit 4), day 36 (visit 8), and day 64 (visit 9). Bars represent geometric mean values, error bars depict 95% confidence intervals, and symbols represent measured values of individual subjects. Statistically significant differences (*p* < 0.05, Friedman test) between groups are indicated above bars.

As shown in Figure 4, for both Cohort 1 and Cohort 2, there was a statistically significant (Friedman test, *p* < 0.05) difference in nasal wash RBD-specific IgA levels from Baseline to each study visit (Visits 4, 8, and 9, on day 15, 36, and 64, respectively); the difference was more pronounced for Cohort 2 (Figure 4D). Nasal wash Spike-specific IgA measurements also showed increased antibody levels from baseline to final study visit, whereas no effect on nucleocapsid IgA responses was observed in nasal washes (see Supplementary S8). Further, there was an apparent decrease in nasal wash RBD-specific IgA levels from Visit 8 to Visit 9 observable in Cohort 1, which was not apparent in Cohort 2, where there was an apparent increase between the same visits. Due to the small sample size of this phase I study, no statistical comparisons between individual time points were performed, and these fluctuations should therefore be interpreted cautiously. Again, there were no observable changes in placebo or OMV groups over the assessment period (see Supplementary S8).

## 4. Discussion

This first-in-human study demonstrates that intranasal administration of detoxified meningococcal OMVs combined with SARS-CoV-2 Spike as antigen is safe, well-tolerated, and immunogenic in healthy adults. Notably, dose-dependent increases in both systemic and mucosal immune responses were observed, with statistically significant enhanced serum IgG antibody levels and nasal wash IgA antibodies in the high-dose group, alongside increases in neutralizing antibodies from baseline to the final visit. No serious adverse events occurred across any study arms, with adverse effects predominantly mild and transient. These findings establish clinical proof-of-concept for the Nm-nOMV platform and provide a strong foundation for further clinical investigation.

This study builds on the preclinical findings that an OMV-based COVID-19 vaccine elicited robust mucosal and systemic immune responses in mice and Syrian hamsters, with significant viral load reduction and lung pathology protection following challenge [6]. The current clinical data confirm key aspects of this preclinical profile, which demonstrate the potential of the Nm-nOMV platform as a vaccine adjuvant with strong mucosal immunostimulatory properties.

Parenteral vaccines for respiratory pathogens have proven highly effective at reducing severe disease and mortality, yet they often show limited efficacy in preventing breakthrough infections and onward transmission, likely because they induce little to no mucosal immunity [19,20]. This limitation is particularly relevant in the context of pandemic preparedness, where controlling transmission at the population level is as critical as protecting individuals from severe disease. Intranasal vaccination offers a compelling alternative by targeting the primary site of pathogen entry by eliciting mucosal secretory IgA that can neutralize pathogens at the epithelial surface before they breach the mucosal barrier, while simultaneously generating systemic immune responses [21]. Beyond these immunological advantages, intranasal administration holds important practical benefits for large-scale pandemic response as it does not require needles or trained healthcare personnel, enabling broader and faster deployment, and it is generally better accepted by people due to the avoidance of injection-related discomfort and anxiety [22]. Despite this potential, intranasal vaccines remain markedly underrepresented in clinical development: of the numerous COVID-19 subunit vaccines evaluated in clinical trials, only a small fraction were designed for intranasal administration [23]. Several intranasal COVID-19 vaccine candidates are currently in pre-clinical and clinical development, including live-attenuated or replication-deficient viral vectors and recombinant protein-based formulations, all aiming to induce mucosal immunity at the primary site of infection [8,9,10]. Compared to these approaches, OMV-based vaccines offer a non-replicating and inherently immunostimulatory platform that can be rapidly engineered and does not require live pathogens or viral vectors [24,25,26]. These features position OMV-based strategies as a complementary approach within the broader landscape of intranasal SARS-CoV-2 vaccine development. Among intranasal vaccine concepts, OMV-based vaccines are well-suited for respiratory pathogens, combining the intrinsic adjuvant properties of bacterial outer membrane vesicles with efficient targeting of the nasal mucosa. Preclinical studies have confirmed that intranasal OMV-based vaccination induces mucosal IgA, systemic IgG, and neutralizing antibody responses comparable to, and in some cases exceeding, those achieved by intramuscular immunization [6,27]. This study adds to this evidence base by confirming safety and tolerability in healthy adults and providing promising immunogenicity outcomes.

As is inherent to a first-in-human Phase I trial, the sample size was not powered for formal immunogenicity testing, and findings should therefore be interpreted as exploratory. The enrollment of exclusively SARS-CoV-2 seropositive adults means that pre-existing immunity likely influenced baseline antibody titers and the magnitude of vaccine-induced responses, thereby limiting direct comparability of seronegative populations. As the study was designed to evaluate the safety and immunogenicity of the selected OMV- Spike formulation rather than the contribution of its individual components, a Spike-only comparator arm was not included. This precludes direct assessment of the contribution of OMVs to the observed immune responses. Preclinical data supports a potential adjuvant role of OMVs; Syrian hamsters immunized with OMV + Spike showed markedly superior protection, with near-complete absence of lung lesions and enhanced reduction in viral load compared to the Spike alone group [6]. These adjuvant effects, however, were not evaluated in the current trial. These effects are likely underpinned by the intrinsic adjuvant activity of OMVs, which is most likely driven by the combined action of multiple outer membrane components, including lipopolysaccharide and lipoproteins, which collectively engage innate immune pathways via TLR2 and TLR4 rather than a single dominant factor or a purely liposomal reservoir effect [5]. Building on these findings, a subsequent Phase II trial adequately powered for formal immunogenicity analyses and including a Spike-only comparator arm would enable a more definitive assessment of the adjuvant contribution of OMVs.

Beyond the immunological profile, the Nm-nOMV platform offers several practical advantages relevant to pandemic preparedness. Real-time stability data support a shelf-life of at least 2.5 years under standard refrigerator conditions in ongoing studies, enabling stockpiling for rapid deployment. Zoonotic pathogens remain a continuous risk for epidemics or pandemics. Spillovers from coronaviruses circulating in the animal population to humans can recur in the future [28]. Novel coronaviruses continue to emerge in the reservoir host and reported cases of MERS-CoV in Europe highlight that the risk of rapid international spread is real [29,30]. The plug-and-play nature of the Nm-nOMV platform enables swift adaptation against such threats, supporting its development as a pan-coronavirus vaccine [31]. Together, these immunological and practical advantages support the rationale for advancing intranasal OMV-based vaccines as a strategy combining robust systemic protection with strong mucosal immunity—a profile particularly relevant for broadly applicable vaccines against rapidly emerging pathogens with pandemic potential.

## 5. Conclusions

Building on previous preclinical findings, this first-in-human phase I trial of the intranasal Nm-nOMV platform combined with SARS-CoV-2 Spike protein in healthy adults confirmed its safety and tolerability, with no serious adverse events and predominantly mild and transient adverse events across all study arms. In addition, exploratory immunogenicity data suggest a potential dose-dependent response to the OMV-Spike vaccine. The consistent safety profile observed across species, combined with encouraging immunogenicity data, provides a robust foundation for advancement to further clinical investigation. Further development of this platform may play a vital role in enhancing global pandemic preparedness.

## 6. Patent

The results of the phase I study are the subject of patent application EP26182309.

## Figures and Tables

**Table 1:** Baseline characteristics of the participants per cohort.

	Cohort 1			Cohort 2		
Characteristic	OMV + Spike (140 μg of OMV and 70 μg of Spike Protein)	OMV (140 μg)	Placebo	OMV + Spike (280 μg of OMV and 140 μg of Spike Protein)	OMV (280 μg)	Placebo
Number of participants	13	3	5	13	3	3
Age (years), mean (s.d.)	42.7 (10.4)	43.7 (12.9)	42.0 (13.7)	47.1 (8.6)	31.3 (13.3)	49.0 (8.7)
Gender, *n* (%)						
Female	7 (53.8%)	1 (33.3%)	2 (40.0%)	11 (84.6%)	2 (66.7%)	1 (33.3%)
Male	6 (46.2%)	2 (66.7%)	3 (60.0%)	2 (15.4%)	1 (33.3%)	2 (66.7%)
Childbearing potential, *n* (%)						
Yes	4 (57.1%)	1 (100%)	2 (100%)	5 (45.5%)	2 (100%)	0
Permanently Sterilized	1 (14.4%)	0	0	0	0	0
Postmenopausal	2 (28.6%)	0	0	6 (54.5%)	0	1 (100%)
BMI (kg m^−2^), mean (s.d.)	24.87 (3.04)	31.00 (0.92)	22.74 (2.37)	26.54 (3.27)	22.03 (3.97)	24.23 (1.40)
Race, *n* (%)						
White	10 (76.9%)	3 (100%)	4 (80.0%)	12 (92.3%)	2 (66.7%)	3 (100%)
Black or African American	0	0	0	0	0	0
Native Hawaiian/Pacific Islander	1 (7.7%)	0	0	0	0	0
Other	2 (15.4%)	0	1 (20%)	1 (7.7%)	1 (33.3%)	0
Ethnicity *n* (%)						
Hispanic or Latino	0	0	0	0	0	0
Not Hispanic or Latino	13 (100%)	3 (100%)	5 (100%)	13 (100%)	3 (100%)	3 (100%)

**Table 2:** Overall summary of treatment-emergent adverse events (TEAEs).

	Cohort 1			Cohort 2		
Number of Participants Reporting at Least 1	OMV + Spike (140 μg of OMV and 70 μg of Spike Protein) (*n* = 13) *X* (%) E	OMV 140 μg (*n* = 3) *X* (%) E	Placebo (*n* = 5) *X* (%) E	OMV + Spike (280 μg of OMV and 140 μg of Spike Protein) (*n* = 13) *X* (%) E	OMV 280 μg (*n* = 3) *X* (%) E	Placebo (*n* = 3) *X* (%) E
Number of TEAEs	11 (84.6%) 31	3 (100%) 10	5 (100%) 11	10 (76.9%) 21	3 (100%) 7	1 (33.3%) 1
Serious TEAE	0	0	0	0	0	0
Severe TEAE	0	0	0	1 (77.7%) 1	0	0
Related TEAE	6 (46.2%) 10	1 (33.3%) 1	4 (80%) 8	5 (38.5%) 8	3 (100%) 5	0
TEAE leading to study withdrawal	1 (7.7%) 1	0	0	0	0	0
TEAE leading to death	0	0	0	0	0	0
AESI	7 (53.8%) 14	1 (33.3%) 2	3 (60%) 6	5 (38.5) 8	2 (66.7%) 2	0
Study drug-related AESI	6 (46.2%) 10	1 (33.3%) 1	3 (60%) 6	5 (38.5%) 7	2 (66.7%) 2	0

Abbreviations: AESI = adverse event of special interest; OMV = outer membrane vesicle; TEAE = treatment-emergent adverse event. Note(s): Relationship to study treatment is defined as AEs deemed at least possibly related to study treatment. Drug withdrawal is defined as any action taken to prohibit further dosing. Treatment-emergent adverse events (TEAEs) are defined as AEs that occurred following the first administration of study medication. If a participant has multiple occurrences of a TEAE, the participant is presented only once in the Participant count (X) column. Occurrences are counted each time in the occurrence count (E) column. Percentages are calculated based on the number of participants in the Safety Population in each treatment group (*n*).

**Table 3:** Frequency of treatment-emergent adverse events (TEAEs) reported by participants in Cohort 1, presented by severity grade. If a participant has multiple occurrences of a TEAE, the participant is presented only once in the Participant count (*n*) column for a given system order class (SOC) and preferred term (PT). Occurrences are counted each time in the occurrence count (E) column. Percentages are calculated based on the number of participants in the safety set in each treatment group (*n*).

Cohort 1									
System Organ Class (SOC) Preferred Term (PT)	OMV + Spike (140 μg of OMV and 70 μg of Spike Protein) (*n* = 13) *n* (%) E			OMV 140 μg (*n* = 3) *n* (%) E			Placebo (*n*= 3) *n* (%) E		
	Grade 1	Grade 2	Grade 3	Grade 1	Grade 2	Grade 3	Grade 1	Grade 2	Grade 3
Respiratory, Thoracic, and Mediastinal Disorders									
Oropharyngeal Pain	3 (23.1%) 3	1 (7.7%) 1	0	0	0	0	1 (20%) 1	0	0
Cough	1 (7.7%) 1	0	0	0	0	0	2 (40%) 2	0	0
Nasal Congestion	2 (15.4%) 2	0	0	1 (33%) 1	0	0	0	0	0
Rhinorrhea	2 (15.4%) 2	0	0	1 (33%) 1	0	0	0	0	0
Throat Irritation	0	0	0	0	0	0	1 (20%) 1	0	0
Upper Respiratory Tract Congestion	1 (7.7%) 1	0	0	0	0	0	0	0	0
Infections and Infestations									
Herpes Simplex	0	0	0	0	0	0	1 (20%) 1	0	0
Nervous System Disorders									
Headache	2 (15.4%) 4	1 (7.7%) 1	0	1 (33.3%) 1	0	0	1 (20%) 1	0	0
Sinus Headache	1 (7.7%) 1	0	0	0	0	0	0	0	0
Skin and Subcutaneous Tissue Disorders									
Erythema	0	0	0	0	0	0	1 (20%) 1	0	0
General Disorders and Administration Site Conditions									
Fatigue	0	0	0	0	0	0	1 (20%) 1	0	0

**Table 4:** Frequency of treatment-emergent adverse events (TEAEs) reported by participants in Cohort 2, presented by severity grade. If a participant has multiple occurrences of a TEAE, the participant is presented only once in the Participant count (X) column for a given system order class (SOC) and preferred term (PT). Occurrences are counted each time in the occurrence count (E) column. Percentages are calculated based on the number of participants in the safety set in each treatment group (*n*).

Cohort 2									
System Organ Class (SOC) Preferred Term (PT)	OMV + Spike (280 μg of OMV and 140 μg of Spike Protein) (*n* = 13) *X* (%) E			OMV 280 μg (*n* = 3) *X* (%) E			Placebo (*n* = 3) *X* (%) E		
	Grade 1	Grade 2	Grade 3	Grade 1	Grade 2	Grade 3	Grade 1	Grade 2	Grade 3
Nervous System Disorders									
Headache	1 (7.7%) 1	1 (7.7%) 2	0	2 (66.7%) 2	0	0	0	0	0
Respiratory, Thoracic, and Mediastinal Disorders									
Cough	1 (7.7%) 2	0	0	0	0	0	0	0	0
Sneezing	1 (7.7%) 1	0	0	0	0	0	0	0	0
Throat Irritation	1 (7.7%) 1	0	0	0	0	0	0	0	0
Skin and Subcutaneous Tissue Disorders									
Night Sweats	0	0	0	1 (33.3%) 2	0	0	0	0	0
Pruritus	1 (7.7%) 1	0	0	0	0	0	0	0	0
General Disorders and Administration Site Conditions									
Fatigue	0	1 (7.7%) 1	0	0	0	0	0	0	0
Hepatobiliary Disorder									
Hyperbilirubinemia	0	0	0	1 (33.3%) 1	0	0	0	0	0
Psychiatric Disorder									
Confusional State	1 (7.7%) 1	0	0	0	0	0	0	0	0

## Data Availability

The original contributions presented in this study are included in the article and Supplementary Materials. Access to the full clinical trial dataset is restricted due to participant privacy obligations and contractual agreements governing the investigational product. Anonymized data, the trial protocol and/or statistical analysis plan may be shared following a reasonable formal data access request directed at the corresponding authors.

## Supplementary Materials

The following supporting information can be downloaded at: https://www.mdpi.com/article/10.3390/vaccines14070575/s1, Supplementary S1: Inclusion and Exclusion Criteria; Supplementary S2: Schedule of Assessments; Supplementary S3: Summary of Protocol Amendments; Supplementary S4: Safety Assessment Parameters; Supplementary S5: Serum SARS-CoV-2 Neutralizing Antibody Concentrations; Supplementary S6: Serum Anti-SARS-CoV-2-antigen (Spike, Receptor Binding Domain (RBD), and Nucleoprotein) IgA Concentration; Supplementary S7: Serum Anti-SARS-CoV-2 (Spike, Receptor Binding Domain (RBD), and Nucleoprotein) IgG Concentrations; Supplementary S8: Nasal Wash Anti-SARS-CoV-2 (Spike, Receptor Binding Domain (RBD), and Nucleoprotein) IgA Concentrations.

## Abbreviations

The following abbreviations are used in this manuscript:

OMV	Outer Membrane Vesicle
PAMP	Pathogen-Associated Molecular Pattern
GCP	Good Clinical Practice
HREC	Human Research Ethics Committee
FDA	Food and Drug Administration
ECG	Electrocardiogram
BP	Blood Pressure
PR	Pulse Rate
BMI	Body Mass Index
FU	Follow-Up
AE	Adverse Event
SAE	Serious Adverse Event
TEAE	Treatment Emergent Adverse Event
NAbs	Neutralizing Antibodies
