# Supplementary material for: A Randomized, Double-Blind, Placebo-Controlled Phase I Study to Evaluate the Safety, Tolerability, and Immunogenicity of an Outer Membrane Vesicle (OMV) Platform-Based Vaccine Administered Intranasally to Healthy Adults
Source: Vaccines (Basel). 2026 Jun 29;14(7):575. doi: 10.3390/vaccines14070575 (PMC13417143; doi:10.3390/vaccines14070575)
Supplement: Supplementary file 1 [file vaccines-14-00575-s001.zip › vaccines-4323043-supplementary.pdf]

Supplementary S1

Inclusion and Exclusion criteria

Inclusion Criteria

Participants who met all of the following inclusion criteria at Screening were eligible to participate:

1. Healthy male or female participants between the ages of 18 and 55 years, inclusive. Healthy was defined as no clinically relevant abnormalities identified by a detailed medical history, a full physical/neurological examination including vital signs (including systolic and diastolic blood pressure [BP], temperature, and pulse rate [PR]), a 12-Lead ECG, and clinical laboratory tests).

2. Participant must have received a vaccination against SARS-CoV-2 or have been exposed to SARS-CoV-2 at least ≥ 4 months prior to the first study dose or shown to be seropositive to IgG by any of the serological tests marketed as Emergency Use Authorization and authorized by the FDA.

3. Negative test for SARS-CoV-2 according to CRU standard methods at first visit (Day 1) prior to dosing.

4. Females must have been non-pregnant and non-lactating and were required to use an acceptable, highly effective contraception in the case of heterosexual intercourse. Highly effective contraception was defined as methods which have a failure rate of less than 1% per year:

a. Established hormonal contraception (oral contraceptive pills [OCPs], long-acting implantable hormones, injectable hormones, and the vaginal ring) with the use of a barrier method/condom 30 days prior to dosing and for at least 90 days after the last intranasal administration.

b. An intrauterine device (IUD) placed 30 days prior to first dosing and for at least 90 days after the last intranasal administration.

c. Documented evidence of surgical sterilization at least 6 months prior to Screening (e.g., bilateral tubal occlusion, complete hyster-ectomy, bilateral salpingectomy, or bilateral oophorectomy) for women.

d. Vasectomy at least 90 days prior to enrolment for men (with appropriate post-vasectomy documentation of the absence of sperm in semen), provided the male partner is a sole partner.

• Women not of childbearing potential must have been postmenopausal for ≥ 12 months. When postmenopausal for < 12 months, postmenopausal status could be confirmed through testing of follicle-stimulating hormone (FSH) levels ≥ 40 IU/L at Screening for amenorrheic female participants, at the discretion of the Investigator, or the participant was considered to be of childbearing potential.

• Women of childbearing potential (WOCBP) must have had a negative pregnancy test at Day 1 and be willing to take additional pregnancy tests as required throughout the study.

• Females who were abstinent from heterosexual inter-course, as their usual practice, were also eligible. Female participants who were exclusively in same-sex relationships are not required to use contraception.

• Participant total abstinence from heterosexual intercourse, if this was their usual practice, for 30 days prior and for 90 days prior after the last study treatment was acceptable. Periodic abstinence (e.g., calendar, ovulation, symptothermal, post-ovulation methods) and withdrawal were not acceptable forms of contraception.

5. Males must have been surgically sterile (> 90 days since vasectomy with appropriate post-vasectomy documentation of the absence of sperm in semen), abstinent as a usual practice, or if engaged in sexual relations with a WOCBP, the participant and his partner must have been surgically sterile (e.g., bilateral tubal occlusion, hysterectomy, bi-lateral salpingectomy, bilateral oophorectomy) or using an acceptable, highly effective double contraceptive method from Screening until study completion, including the Follow-up period. Acceptable methods of double contraception included the use of condoms in addition to the use of an effective contraceptive for the female partner that included: OCPs, long-acting implantable hormones, injectable hormones, a vaginal ring, or an IUD. Participants with same-sex partners (abstinence from penile-vaginal intercourse) were eligible when this was their preferred and usual lifestyle. Males could not donate sperm for at least 90 days after Nm-nOMV mixed with antigen administration.

6. Body mass index (BMI) between 18.0 to 32.0 kg/m2, inclusive; and a total body weight ≥ 50.0 kg for males and ≥ 45.0 kg for females.

7. Written or electronic informed consent from the patient prior to any study procedures in a manner approved by HREC.

8. Willing and able to comply with the scheduled visits, confinement period, treatment plan, laboratory tests, and other trial procedures and requirements.

<u>Exclusion Criteria</u>

Participants who met any of the following exclusion criteria at Screening were not eligible to participate:

1. Evidence or history of medical conditions which were unstable, or under investigations currently, defined as major changes to management/medication or surgery/hospitalization in the last 12 months, including hematological (including clotting disorders), renal, endocrine, pulmonary, gastrointestinal, cardiovascular, hepatic, psychiatric, neurologic, or allergic disease (including drug allergies, and untreated sea-sonal allergies at time of dosing).

2. History of any clinically significant autoimmune disorder (such as Guillain-Barré syndrome).

3. History of febrile illness within 14 days prior to the first dose.

4. Confirmed SARS-CoV-2 infection within the last 4 months prior to study enrolment. Known exposure to another person with SARS-CoV-2 infection within the last 14 days prior to study enrolment.

5. Vaccination with a live vaccine within the 4 weeks prior to study enrolment or any non-live vaccination within the 2 weeks prior to study enrolment, or that was planned during study participation. Live and/or non-live vaccines during the Follow-up period (between Visit 9 and Visit 10) are allowed.

6. Vaccination against N. meningitidis (type B).

7. History of frequent epistaxis (defined as a weekly occurrence, or more frequently).

8. Evidence of a deviated septum, or other nasal abnormality, which may have impeded the ability for intranasal administration of any study medication.

9. History of, or current positive results for, any of the following serological tests: human immunodeficiency virus (HIV), hepatitis B surface antigen (HBsAg), hepatitis B core antibody (HBcAb), or hepatitis C antibody (HCVAb).

For Hepatitis B Screening:

− Participants who were HBsAg positive will be excluded.

− Participants who had negative HBsAg, positive HBcAb, and negative HBsAb were to be excluded.

− Participants who had negative HBsAg, negative HBcAb, and positive HBsAb and provided documentation (participant attestation of vaccination status could suffice if documentation was not available) of prior hepatitis B vaccination are eligible for the study.

For Hepatitis C Screening:

− Participants who were positive for HCVAb were excluded.

10. Active malignancy or a history of malignancy in the previous 5 years, except for adequately treated or excised non-metastatic basal cell or squamous cell cancer of the skin or adequately treated cervical carcinoma-in-situ.

11. Documented history of alcohol, cocaine, or IV drug abuse within 6 months of study enrolment and any sign of alcohol use in the 24 hours prior to the visit.

12. Treatment with another IP or participated in another interventional clinical trial within 90 days, preceding study enrolment.

13. Use of prescription and non-prescription intranasal drug within 7 days prior to first dose of intranasal administration and throughout the study, until 1 month after the last intranasal administration.

14. Screening supine BP ≥ 155 mmHg (systolic) or ≥ 95 mmHg (diastolic), following at least 5 minutes of supine rest. If BP was ≥ 155 mmHg (systolic) or ≥ 95 mmHg (diastolic), the BP was to be repeated 2 more times with the participant at supine rest

and the average of the 3 BP values was to be used to determine the participants' eligibility.

15. Screening 12-Lead ECG following at least 5 minutes of supine rest demonstrating a Fridericia corrected QT (QTcF) interval > 450 msec (for males) or > 470 msec (for females) or a QRS interval ≥ 120 msec. If QTcF exceeded 450 msec for men or 470 msec for females, or QRS exceeded ≥ 120 msec, the ECG was to be repeated 2 more times and the average of the 3 QTcF (or QRS) values was to be used to determine the participants' eligibility.

16. Participants with clinically significant abnormalities (as determined by the Investigator) in clinical laboratory tests at Screening, as assessed by the study-specific clinical laboratory. A single repeat test could be conducted if deemed necessary. Exception could be granted at the discretion of the Investigator where no confounding factors were expected to impact the safety of the participants or the integrity of the study

17. Pregnant, lactating, or planning to become pregnant (self or partner) at any time during the study, or 90 days after last intranasal administration.

18. Blood or plasma donation of approximately 500 mL or more within 90 days prior to the first intranasal administration.

19. Previous history of intolerance or hypersensitivity to any component of the IP formulation.

20. Participants who had previously experienced a Grade 3 or higher AE to receipt of a SARS-CoV-2 vaccination.

21. Participants who were investigational site staff members directly involved in the conduct of this study and their family members, site staff otherwise supervised by the Investigator, and participants who were employees of the Sponsor or were agents of the Sponsor.

22. Any other reason, criteria that could interfere with the interpretation of study results or, in the judgement of the Investigator, could make the participant inappropriate for entry into this study.

Supplementary S2

Table S1. Schedule of Assessments.

**Visit Number**	**Screening**	**1**	**2**	**3**	**4**	**5**	**6**	**7**	**8**	**9**	**Unplanned**	**10**
**Visit Description**	**Screening**	**Intranasal Dose 1**	**3-Day FU Visit (Dose 1)**	**1-Week Safety Call (Dose 1)**	**2-Week FU Visit (Dose 1)**	**Intranasal Dose 2**	**3-Day FU Visit (Dose 2)**	**1-Week Safety Call (Dose 2)**	**2-Week FU Visit (Dose 2)**	**1-Month FU Visit (Dose 1)**	**Unscheduled Visit[16]**	**6-Month FU Call (Dose 2)**
Informed consent	X											
Inclusion/ Exclusion	X	X										
Demographics	X											
Medical History	X	X										
Prior/Con. Medication	X	X	X	X	X	X	X	X	X	X	X	X
Physical/Neurological Exam, Height, and Weight	X[1]									X	(X)	
Serum FSH	X											
Pregnancy Test[2]	X	X				X					(X)	
Serology[3]	X											
SARS-COV-2 Test[4]	X	X				X				X	(X)	
Urine Drug Screen/Breath Alcohol Test[5]	X	X	X		X	X	X		X	X	(X)	
Randomization		X				X						
Dose Administration[6]		X				X						
12-Lead ECG	X	X[7]	X			X[7]	X			(X)		

Vital Signs (BP [Systolic and Diastolic], PR, Temperature)	X	X[8]	X		X	X[8]	X		X	X	(X)	
Pulse Oximetry	X	X[9]	X		X	X[9]	X		X	X	(X)	
Clinical Laboratory Samples[10]	X	X	X			X	X			X	(X)	
Immunogenicity Blood Samples	X[11,12]	X			X	X			X	X		
Nasal Washes	X[12]				X				X	X		
Samples Cellular Response		X			X	X			X	X		
Local Tolerability Assessment[13]		X[14]	X		X	X[14]	X		X		(X)	
Participant Diary[15]		X					X					
Adverse Events	X	X	X	X	X	X	X	X	X	X	X	X

Abbreviations: AE = adverse event; BP = blood pressure; CRU = clinical research unit; ECG = echocardiogram; FSH = follicle-stimulating hormone; FU = Follow-up; HIV = human immunodeficiency virus; PR = pulse rate; SARS-CoV-2 = severe acute respiratory syndrome corona virus 2

[1] Height and weight were only measured at Screening. A full physical/neurological examination was performed at Screening and at the 1-Month post final administration visit. Unscheduled symptom-directed physical examinations could be performed at the discretion of the Investigator or designee.

[2] A serum pregnancy test was performed at Screening and at all other times a urine pregnancy test was conducted. If a urine test was positive, at the Investigator's discretion, a serum test could be performed to confirm the pregnancy status. If the serum test was negative, the participant could still be considered eligible. The pregnancy test was to serve as a checkpoint event, and a positive test excluded the participant from further participation in the study.

[3] Serology assessments included screening for HIV, Hepatitis B, and C.

[4] A SARS-CoV-2 test was performed according to CRU standard methods. The SARS-CoV-2 test served as a checkpoint event and a positive test excluded the participant from further participation in the study.

[5] A urine drug screen/alcohol breath test was performed at Screening and prior to first and second dosing. Additional alcohol breath tests were performed at each CRU visit.

[6] Drug administration was performed on the morning of each dosing day.

[7] On dosing days, ECG was performed predose (within 120 minutes of dose administration), as well as at approximately 60 minutes postdose.

[8] On dosing days, vital signs were performed predose (within 120 minutes of dose administration), as well as at approximately 60 minutes postdose.

[9] On dosing days, pulse oximetry was performed predose (within 120 minutes of dose administration), as well as at approximately 60 minutes postdose.

[10] Clinical laboratory samples consisted of hematology, biochemistry, and urinalysis.
[11] Immunogenicity samples at Screening were used for the confirmation of pre-existing SARS-CoV-2 antibodies.
[12] Immunogenicity samples and nasal washes collected at Screening were collected at least 3 days prior to Visit 1. If possible, the nasal wash was done on a different day as the SARS-CoV-2 test. If this was not logistically possible, the SARS-CoV-2 test was to be conducted prior to the nasal wash at Screening.
[13] Additional ad hoc assessments could also be performed in the event of an AE, or at the discretion of the Investigator or designee. A local tolerability assessment consisted of an inspection of the nose and throat of the participant, internally and externally.
[14] On dosing days, local tolerability assessments were performed predose (within 120 minutes of dose administration), as well as at approximately 60 minutes postdose.
[15] Participants were provided with a paper diary for a period of 2 weeks following each dose administration. The diary consisted of a set of defined questions querying the participant's experiences and any reactions to the study treatment, as well as any concomitant medications taken. If the participant had experienced any adverse reactions (such as headache, fever, fatigue, etc.), they were instructed to describe the event, the time it occurred, and when (and if) the event resolved.
[16] At the discretion of the Investigator, participants could be invited to return to the CRU for an unscheduled Follow-up visit. At minimum the visit was to consist of a review of concomitant medications and AEs, but all other activities were to be determined by the Investigator on a case-by-case basis.

Supplementary S3

Table S2. Summary of Changes Made per Protocol Amendment.

**Protocol amendment**	**Effective date**	**Main Changes Made and Rationale**	**# of participants enrolled to protocol version**
Version 1.0	28-Jul-22	Not applicable	0 participants.
Version 2.0	2-Nov-22	• Minor inconsistencies noted between Schedule of Assessments and Study Schedule; corrections made to avoid confusion.<br>• Visit windows added to follow-up visits (Day 4, 22, 25) to improve flexibility.<br>• Nasal wash draining procedure modified to align with Gritzfeld *et al.* (2011).<br>• Secondary endpoints revised to better define days of analyses and align with development program goals.<br>• Inter-dose period redefined from 'approximately' to 'at least' 3 weeks to ensure conformity between study practice and real-world application.<br>• Terminology for study dates revised to aid interpretation.<br>• Eligibility criteria clarified to remove ambiguity.<br>• Immunomodulatory medications added to prohibited list to reduce impact on investigational product efficacy.<br>• Urine drug screen added prior to first dosing to improve subject compliance and data quality.	4 participants in Cohort 1
Version 3.0	21-Dec-22	• Subject replacement criteria revised: subjects not completing two doses (rather than any dose) are replaced, to prevent unintended reduction of the study population for non-safety reasons.<br>• Pre-dose assessment window harmonized from 60 to 120 minutes in the Schedule of Assessments, following clinical site feedback.	16 participants in Cohort 1.<br>19 participants in Cohort 2.
Version 4.0	26-May-23	• Primary endpoint revised; distinction between solicited and unsolicited adverse effects (AEs) removed, as non-specific AE collection is preferred approach.<br>• Additional AESIs included based on ongoing review of SARS-CoV-2 clinical development programs.<br>• Non-live vaccinations restriction clarified: live and non-live vaccines permitted during the follow-up period between Visit 9 and Visit 10.<br>• Minor discrepancy in AE recording between Schedule of Assessments and corresponding text (Section 13.2) corrected.	1 participant in Cohort 1.

Supplementary S4

Table S3. Safety Assessments.

<table>
<tr><th>Safety Assessments</th><th colspan="2">Description</th></tr>
<tr><td>Pregnancy test</td><td colspan="2">Serum or urine test for beta-human chorionic gonadotropin (β-hCG), as indicated for women of childbearing potential.</td></tr>
<tr><td>Physical/Neurological examination</td><td colspan="2">Complete and symptom-directed physical examinations were to be performed by a licensed physician according to the conduct of CRU standard procedures.</td></tr>
<tr><td>Vital signs</td><td colspan="2">Included temperature, systolic and diastolic BP, and PR. Participants were to be resting for at least 5 minutes in a supine position</td></tr>
<tr><td rowspan="2">Hematology (local)</td><td>• Hemoglobin (HGB)
• Hematocrit (HCT)
• Erythrocytes (RBC)</td><td>• Platelets (PLAT)
• Leukocytes with differential</td></tr>
<tr><td colspan="2">Differential (absolute) counts: eosinophils [ESN], neutrophils [NEUT], basophils [BASO], lymphocytes [LYM], and monocytes [MONO])</td></tr>
<tr><td>Biochemistry (local)</td><td>• C-reactive protein (CRP)
• Blood urea nitrogen/urea (BUN)
• Creatinine (CREAT)
• Total bilirubin (BILI) and direct bilirubin (BILIDIR)
• Urate (URATE)
• Albumin (ALB)
• Globulin (GLOBUL)
• Alkaline phosphatase (ALP)
• Creatine kinase (CK)
• Troponin I (TROPI)
• Aspartate aminotransferase (AST)</td><td>• Alanine aminotransferase (ALT)
• Gamma-glutamyltransferase (GGT)
• Glucose (GLU) (fasting labs only)
• Sodium (NA)
• Potassium (K)
• Calcium (CA)
• Chloride (CL)
• Phosphate (PHOS)
• Bicarbonate (BICARB)</td></tr>
<tr><td>Urinalysis (local)</td><td>• pH (PH)
• Specific gravity (SPGRAV)
• Creatinine (CREATININE)
• Protein (PROT)
• Glucose (GLUC)
• Ketones (KETONES)</td><td>• Total bilirubin (BILI)
• Occult blood (OCCBLD)
• Nitrite (NITRITE)
• Urobilinogen (UROBIL)
• Leukocytes (WBC)</td></tr>
<tr><td>12-lead ECG (local)</td><td colspan="2">Heart rate, PR, QRS, QT, QTc intervals, and overall assessment. A single ECG was to be performed on Day -1 and triplicate ECGs were to be performed at Screening, [predose, and at 1 hour].</td></tr>
</table>

Abbreviations: BP = blood pressure; CRU = clinical research unit; ECG = electrocardiogram; HCG = human chorionic gonadotrophin; PR = pulse rate

Supplementary S5

Table S4. Serum SARS-CoV-2 Nabs concentration for Cohort 1 and 2.

**Serum SARS-CoV-2 Nabs Concentration (IU/mL)**	**Visit**	**Cohort 1**			**Cohort 2**		
		**OMV + Spike (*n*=12)** Mean (SD)	**OMV (*n*=3)** Mean (SD)	**Placebo (*n*=3)** Mean (SD)	**OMV + Spike (*n*=12)** Mean (SD)	**OMV (*n*=3)** Mean (SD)	**Placebo (*n*=3)** Mean (SD)
Actual value	Baseline[1]	1280 (1290)	1660 (1020)	1450 (766)	882 (561)	195 (68.7)	1590 (894)
	Visit 4	1280 (1150)	1530 (818)	1720 (1180)	947 (589)	176 (109)	1470 (989)
	Visit 5	1390 (1450)	1810 (1150)	1690 (699)	1050 (776)	181 (81.1)	1370 (602)
	Visit 8	1130 (1080)	1410 (781)	999 (798)	1220 (1110)	211 (124)	1270 (390)
	Visit 9	1330 (1460)	1640 (816)	1550 (784)	1230 (1120)	205 (48.8)	1610 (864)

[1] Baseline is defined as the last available valid, non-missing observation for each subject prior to first study drug administration.

Supplementary S6

Table S5. Anti-SARS-CoV-2 Spike IgA for Cohort 1 and 2.

Anti-SARS-CoV-2 **Spike IgA**	**Visit**	**Cohort 1**			**Cohort 2**		
		**OMV + Spike (*n*=12)** Mean (SD)	**OMV (*n*=3)** Mean (SD)	**Placebo (*n*=3)** Mean (SD)	**OMV + Spike (*n*=12)** Mean (SD)	**OMV (*n*=3)** Mean (SD)	**Placebo (*n*=3)** Mean (SD)
Actual value	Baseline[1]	2240 (2400)	759 (864)	639 (459)	1080 (1220)	290 (465)	1800 (1080)
	Visit 4	2460 (2840)	766 (870)	958 (1050)	1470 (1300)	267 (432)	1500 (869)
	Visit 5	2790 (3300)	727 (798)	874 (919)	1340 (994)	230 (367)	1660 (1030)
	Visit 8	2880 (3450)	832 (1000)	921 (1010)	1510 (1370)	255 (409)	768 (705)
	Visit 9	2750 (3360)	673 (643)	895 (1030)	1590 (1300)	188 (297)	1440 (885)

[1] Baseline is defined as the last available valid, non-missing observation for each subject prior to first study drug administration.

Table S6. Anti-SARS-CoV-2 RBD IgA for Cohort 1 and 2.

Anti-SARS-CoV-2 **RBD IgA**	**Visit**	**Cohort 1**			**Cohort 2**		
		**OMV + Spike (*n*=12)** Mean (SD)	**OMV (*n*=3)** Mean (SD)	**Placebo (*n*=3)** Mean (SD)	**OMV + Spike (*n*=12)** Mean (SD)	**OMV (*n*=3)** Mean (SD)	**Placebo (*n*=3)** Mean (SD)
Actual value	Baseline[1]	2460 (3050)	750 (754)	843 (838)	975 (777)	324 (523)	2470 (1530)
	Visit 4	2760 (3540)	720 (697)	1110 (1310)	1310 (889)	295 (478)	2090 (1270)
	Visit 5	3020 (3770)	641 (564)	1470 (1950)	1240 (893)	265 (427)	2240 (1380)
	Visit 8	3000 (4330)	747 (778)	1470 (1970)	1460 (1190)	298 (480)	1360 (1590)
	Visit 9	3170 (4730)	612 (425)	1250 (1690)	1540 (1140)	222 (353)	1930 (111)

[1] Baseline is defined as the last available valid, non-missing observation for each subject prior to first study drug administration.

Table S7. Anti-SARS-CoV-2 Nucleoprotein IgA for Cohort 1 and 2.

Anti-SARS-CoV-2 **Nucleoprotein IgA**	**Visit**	**Cohort 1**			**Cohort 2**		
		**OMV + Spike (*n*=12)** Mean (SD)	**OMV (*n*=3)** Mean (SD)	**Placebo (*n*=3)** Mean (SD)	**OMV + Spike (*n*=12)** Mean (SD)	**OMV (*n*=3)** Mean (SD)	**Placebo (*n*=3)** Mean (SD)
Actual value	Baseline[1]	59.5 (64.6)	25.6 (15.9)	126 (151)	37.5 (26.2)	10.7 (4.22)	109 (55.9)
	Visit 4	47.9 (50.6)	26.5 (19.7)	136 (150)	77.4 (129)	9.91 (3.89)	88.9 (42.1)
	Visit 5	49.1 (53.1)	22.7 (10.6)	118 (113)	59.3 (76.9)	9.40 (3.16)	107 (56.2)
	Visit 8	43.8 (53.8)	26.0 (17.6)	115 (108)	40.5 (29.0)	10.2 (3.72)	92.5 (40.4)
	Visit 9	47.4 (53.7)	30.8 (26.9)	107 (99.5)	43.8 (28.1)	9.15 (2.05)	93.1 (42.9)

[1] Baseline is defined as the last available valid, non-missing observation for each subject prior to first study drug administration.

Supplementary S7

Table S8. Anti-SARS-CoV-2 Spike IgG for Cohort 1 and 2.

Anti-SARS-CoV-2 **Spike IgG**	**Visit**	**Cohort 1**			**Cohort 2**		
		**OMV + Spike (*n*=12)** Mean (SD)	**OMV (*n*=3)** Mean (SD)	**Placebo (*n*=3)** Mean (SD)	**OMV + Spike (*n*=12)** Mean (SD)	**OMV (*n*=3)** Mean (SD)	**Placebo (*n*=3)** Mean (SD)
Actual value	Baseline[1]	2250 (2080)	2720 (3660)	2750 (2330)	1880 (1290)	434 (314)	3330 (2510)
	Visit 4	2320 (1820)	2480 (3290)	3190 (2670)	2150 (1540)	401 (297)	2800 (2060)
	Visit 5	2460 (1970)	2490 (3320)	3160 (2680)	2310 (1740)	380 (273)	3100 (2530)
	Visit 8	2350 (2010)	2930 (4120)	2880 (2400)	2310 (1800)	396 (289)	3040 (2470)
	Visit 9	2350 (1950)	2140 (2640)	3030 (2540)	2590 (2040)	328 (226)	2780 (2220)

[1] Baseline is defined as the last available valid, non-missing observation for each subject prior to first study drug administration.

Table S9. Anti-SARS-CoV-2 RBD IgG for Cohort 1 and 2.

Anti-SARS-CoV-2 **RBD IgG**	**Visit**	**Cohort 1**			**Cohort 2**		
		**OMV + Spike (*n*=12)** Mean (SD)	**OMV (*n*=3)** Mean (SD)	**Placebo (*n*=3)** Mean (SD)	**OMV + Spike (*n*=12)** Mean (SD)	**OMV (*n*=3)** Mean (SD)	**Placebo (*n*=3)** Mean (SD)
Actual value	Baseline[1]	4840 (4100)	5840 (7850)	5720 (4800)	3420 (2240)	547 (493)	7050 (5120)
	Visit 4	4410 (3610)	5080 (6790)	7250 (6460)	4080 (3080)	501 (463)	5960 (4310)
	Visit 5	4660 (3960)	5020 (6680)	7330 (6980)	4290 (3320)	459 (386)	6440 (5140)
	Visit 8	4400 (4120)	5840 (8220)	6630 (6090)	4200 (3230)	492 (445)	6020 (4480)
	Visit 9	4470 (4060)	4370 (5350)	6520 (5790)	4750 (3480)	373 (300)	5710 (4210)

[1] Baseline is defined as the last available valid, non-missing observation for each subject prior to first study drug administration.

Table S10. Anti-SARS-CoV-2 Nucleoprotein IgG for Cohort 1 and 2.

Anti-SARS-CoV-2 **Nucleoprotein IgG**	**Visit**	**Cohort 1**			**Cohort 2**		
		**OMV + Spike (*n*=12)** Mean (SD)	**OMV (*n*=3)** Mean (SD)	**Placebo (*n*=3)** Mean (SD)	**OMV + Spike (*n*=12)** Mean (SD)	**OMV (*n*=3)** Mean (SD)	**Placebo (*n*=3)** Mean (SD)
Actual value	Baseline[1]	77.8 (179)	11.5 (14.2)	79.0 (125)	36.9 (63.6)	5.62 (2.15)	106 (59.8)
	Visit 4	52.7 (102)	13.3 (8.25)	70.8 (106)	91.0 (165)	4.88 (2.30)	89.3 (53.7)
	Visit 5	42.2 (72.8)	12.8 (9.14)	60.0 (86.8)	79.2 (146)	4.76 (2.68)	97.1 (72.9)
	Visit 8	31.7 (53.1)	15.3 (9.46)	53.7 (79.6)	58.5 (90.3)	6.51 (2.33)	88.9 (65.4)
	Visit 9	26.2 (37.4)	10.6 (6.34)	51.9 (78.0)	42.0 (63.8)	5.94 (2.84)	91.6 (78.9)

[1] Baseline is defined as the last available valid, non-missing observation for each subject prior to first study drug administration.

Supplementary S8

Table S11. Nasal Wash Anti-SARS-CoV-2 Spike IgA for Cohort 1 and 2.

Nasal Wash Anti-SARS-CoV-2 **Spike IgA**	**Visit**	**Cohort 1**			**Cohort 2**		
		**OMV + Spike (*n*=12)** Mean (SD)	**OMV (*n*=3)** Mean (SD)	**Placebo (*n*=3)** Mean (SD)	**OMV + Spike (*n*=12)** Mean (SD)	**OMV (*n*=3)** Mean (SD)	**Placebo (*n*=3)** Mean (SD)
Actual value	Baseline[1]	24.3 (606)	6.34 (5.37)	4.55 (2.62)	14.0 (19.8)	1.66 (2.76)	11.0 (1.23)
	Visit 4	17.6 (15.9)	11.3 (12.9)	6.46 (1.56)	37.8 (68.2)	1.13 (1.81)	15.4 (14.8)
	Visit 8	741 (2520)	4.51 (3.09)	6.69 (3.64)	45.0 (76.2)	1.54 (2.60)	41.5 (62.7)
	Visit 9	15.3 (27.9)	4.80 (4.33)	6.53 (6.55)	27.8 (22.9)	0.643 (1.00)	10.1 (5.10)

[1] Baseline is defined as the last available valid, non-missing observation for each subject prior to first study drug administration.

Table S12. Nasal Wash Anti-SARS-CoV-2 RBD IgA for Cohort 1 and 2.

Nasal Wash Anti-SARS-CoV-2 **RBD IgA**	**Visit**	**Cohort 1**			**Cohort 2**		
		**OMV + Spike (*n*=12)** Mean (SD)	**OMV (*n*=3)** Mean (SD)	**Placebo (*n*=3)** Mean (SD)	**OMV + Spike (*n*=12)** Mean (SD)	**OMV (*n*=3)** Mean (SD)	**Placebo (*n*=3)** Mean (SD)
Actual value	Baseline[1]	8.87 (14.9)	9.76 (9.97)	6.60 (4.69)	22.0 (44.2)	2.24 (3.27)	17.4 (3.24)
	Visit 4	23.9 (24.7)	8.93 (7.77)	11.5 (8.16)	31.3 (60.9)	1.97 (2.15)	25.0 (19.7)
	Visit 8	16.2 (19.3)	6.68 (4.10)	11.7 (8.64)	46.3 (69.4)	1.81 (2.79)	54.6 (78.9)
	Visit 9	25.7 (53.5)	5.86 (4.84)	4.86 (4.20)	31.2 (21.8)	0.773 (0.994)	16.1 (8.96)

[1] Baseline is defined as the last available valid, non-missing observation for each subject prior to first study drug administration.

Table S13. Nasal Wash Anti-SARS-CoV-2 Nucleoprotein IgA for Cohort 1 and 2.

Nasal Wash Anti-SARS-CoV-2 **Nucleoprotein IgA**	**Visit**	**Cohort 1**			**Cohort 2**		
		**OMV + Spike (*n*=12)** Mean (SD)	**OMV (*n*=3)** Mean (SD)	**Placebo (*n*=3)** Mean (SD)	**OMV + Spike (*n*=12)** Mean (SD)	**OMV (*n*=3)** Mean (SD)	**Placebo (*n*=3)** Mean (SD)
Actual value	Baseline[1]	2.77 (3.46)	0.603 (0.100)	1.35 (1.55)	3.99 (3.90)	0.897 (0.764)	3.38 (2.91)
	Visit 4	5.93 (9.00)	0.817 (0.458)	2.26 (2.48)	4.68 (5.58)	1.01 (0.603)	3.38 (2.97)
	Visit 8	2.42 (4.06)	0.627 (0.395)	1.66 (1.42)	4.53 (6.54)	1.14 (1.41)	3.00 (2.13)
	Visit 9	2.12 (2.60)	0.750 (0.668)	1.10 (0.819)	2.97 (4.49)	0.563 (0.456)	3.74 (4.05)

[1] Baseline is defined as the last available valid, non-missing observation for each subject prior to first study drug administration.
